# Funding for Reproductive Health in Conflict and Post-Conflict Countries: A Familiar Story of Inequity and Insufficient Data

**DOI:** 10.1371/journal.pmed.1000093

**Published:** 2009-06-09

**Authors:** Paul B. Spiegel, Nadine Cornier, Marian Schilperoord

**Affiliations:** 1 Office of the United Nations High Commissioner for Refugees, Geneva, Switzerland; 2 Office of the United Nations High Commissioner for Refugees, Nairobi, Kenya

## Abstract

Paul Spiegel and colleagues discuss a new study that examines funding for sexual and reproductive health programs in conflict-affected low-income countries.

**Linked Research Article**

This Perspective discusses the following new study published in *PLoS Medicine*:

Patel P, Roberts B, Guy S, Lee-Jones L, Conteh L (2009) Tracking Official Development Assistance for Reproductive Health in Conflict-Affected Countries. PLoS Med 6(6): e1000090. doi:10.1371/journal.pmed.1000090

Preeti Patel and colleagues report inequity in the disbursement of official development assistance for reproductive health between countries affected by conflict and those unaffected.

## Context

Reproductive health (RH) and related needs during conflict and post-conflict situations are massive, acute, and complex to meet [1]–[4]. RH indicators for conflict-affected and post-conflict countries are worse than for least-developed countries (LDCs) not directly affected by conflict. Despite studies showing that funding for sexual and reproductive health programmes have consistently not met agreed-upon financial targets, little is known about the actual RH funding required to meet these needs in conflict-affected countries. A new study by Preeti Patel and colleagues published in this issue of *PLoS Medicine* addresses this knowledge gap [5]. This study is important because it attempts to quantify the direct and indirect RH disbursements to conflict-affected countries compared with overall official development assistance (ODA) by country, donor, and different RH activities. It also examines RH disbursements and ODA to non-conflict-affected LDCs.

## The New Study

The authors' main source of data was disbursements from the Creditor Reporting System (CRS) from 2003–2006, which covers approximately 90% of all ODA, including humanitarian aid, to developing countries, including conflict and post-conflict countries. The CRS includes bilateral donors as well as multilateral agencies such as the Global Fund to Fight AIDS, Tuberculosis and Malaria (GFATM; http://www.theglobalfund.org/), the World Bank, and some United Nations (UN) agencies. In order to include other important UN agencies that do not report to the CRS, disbursements from the Financial Tracking System were also included.

The study showed that an average of US$20.8 billion in total ODA was disbursed annually to the 18 conflict-affected countries between 2003 and 2006. An annual average of US$509.3 million (2.4%) was allocated to RH (which included HIV and AIDS funding), representing an average of US$1.30 disbursed per capita per year. There was inequity among countries regarding per capita RH disbursements when compared with per capita gross domestic product and RH outcomes. When only LDCs were examined, the 36 non-conflict-affected countries received 53.3% higher per capita RH expenditures than the 15 conflict-affected LDCs (US$2.30 versus US$1.50 per capita per year), despite worse RH outcomes, except HIV prevalence, for the conflict-affected countries. Overall, 9% of ODA was disbursed for RH activities for non-conflict-affected LDCs compared with 4% for conflict-affected LDCs.

When direct and indirect RH activities were analysed per category, direct RH funding constituted 60.5% of the overall RH funding, with HIV and AIDS composing 76.5% of the direct RH funding and almost half (46.3%) of the overall RH funding. There was a large increase in RH disbursements from 2003 to 2006 to the 18 conflict-affected countries (a 79.9% increase) compared with overall ODA funding (a 22.9% increase) to these countries, but this was due largely to an increase in HIV and AIDS funding. The ODA for the other direct RH funding actually decreased by 35.9% over the same time period.

Bilateral donors provided the largest proportion of average annual RH ODA (67.4%), with the United States Government providing 41.9% followed by the United Kingdom at 7.6% between 2003–2006 for the conflict-affected countries. Most of this US funding was for HIV and AIDS through the US President's Emergency Plan for AIDS Relief (http://www.pepfar.gov/). The multilateral agencies provided 32.6% of the average annual RH ODA with the GFATM providing 12% and the UN agencies 10.6%.

## Strength and Limitations of the Study

To the best of our knowledge, this is the first systematic study to track ODA funding for RH activities in conflict-affected countries using the CRS and Financial Tracking System databases. The authors use clear case definitions, take into account the possibility of double counting in the databases, use disbursement as opposed to commitment of funds, take into account inflation and exchange rates, and divide RH interventions into direct and indirect activities.

All such studies have limitations. Databases are only as good as the quality of data they provide; poor reporting by bilateral and multilateral organisations as well as misclassification can occur, and these weaknesses would be difficult to document. As the authors acknowledge, the data do not include national government expenditure on health from national revenues. The databases do not include aid from private philanthropic organisations such as the Bill & Melinda Gates Foundation, which are big players in development assistance. Although disbursement is better than commitment of funds for assessing funding from donors, actual expenditures of funds by country is a very important outcome when comparing impact indicators as well as capability of countries to absorb funds and spend them effectively. Monitoring this actual expenditure is especially important in conflict-affected and post-conflict countries, which often lack infrastructure, technical expertise, and human resources. The percent allocation of funds used for indirect RH activities are acceptable estimates but are still subjective and could be challenged. Finally, according to the definition used for conflict-affected countries, nearly half of the 18 countries were not in active conflict but rather in a post-conflict setting during 2003 to 2006.

## Health and Policy Implications

Given the definition that Patel and colleagues used for conflict-affected countries, their findings are also applicable to most post-conflict countries. This broadens the policy implications of their recommendations. However, the needs and ability to implement programmes are often different between the two settings. In general, achieving minimum essential services is the priority in acute conflict settings when humanitarian space (defined as a neutral zone where international aid agencies can safely and impartially work in an area in which armed conflict is occurring) is limited [1]–[3],[6]. In post-conflict settings, services can be expanded and become more comprehensive according to the specific context of the situation. Vulnerabilities and risks, such as HIV, may also differ between the two settings [7].

In Patel and colleagues' study, the amount of per capita RH funds that donors disbursed does not appear to be related to the RH needs of the countries. Other than HIV prevalence, which has been studied elsewhere [7], conflict and post-conflict LDCs generally have worse RH indicators than non-conflict LDCs—yet they received *less* RH per capita funding. Furthermore, among conflict and post-conflict LDCs, the RH per capita funding was not associated with the severity of indicators. The authors of the study offer clear explanations for why such funding is inequitable, including geopolitical and historical considerations, governance, security and absorptive capacity limitations, and a longer time horizon to obtain results. However, the resulting inequity, although not necessarily surprising, is concerning and clearly has detrimental effects for populations living in conflict and post-conflict settings.

Some have argued that HIV and AIDS funding has increased to the detriment of other sectors [8]. We do not support this point of view. Although direct RH funding for non–HIV and AIDS activities decreased during this time, HIV and AIDS funding increased. Funds for HIV and AIDS are generally used in the broad sense and would likely have benefited both the other direct RH activities as well as the indirect activities [9]. When the needs are so great, one should not try to reduce funding for one type of activity but rather ensure that overall funding increases and that there is complementarity and integration of interventions.

## The Future

There are insufficient data on the actual RH needs and the associated funding required in conflict and post-conflict countries. A comprehensive analysis using standardised methodology to allow for comparability needs to be undertaken to quantify these needs, their costs, and the resources required to fulfil the different needs according to the different phases of conflict and recovery.

These data can then be used to make equitable and evidence-based decisions according to need. Then the practical and contextual issues mentioned above regarding donor interests and the ability of the countries to effectively implement that aid must be considered. Coordination of aid by donors and recipients in these countries is paramount. To encourage such a comprehensive analysis, advocacy should be directed towards the key donors who contribute the majority of RH funds to these countries; these include the governments of the US and UK as well as the GFATM and the UN agencies.

As the authors state, investigation into distribution patterns of RH ODA to recipient governments, non-governmental organisations, and other agencies needs to be examined to guide effective donorship and programmes in the future. Furthermore, documentation of how HIV funds have been used to cover the other direct and indirect RH activities is needed to better understand how funds designated for certain activities interact to achieve RH outcomes.

If the world is to meet the Millennium Development Goals, especially those related to child mortality, maternal health, and HIV/AIDS, then RH issues related to conflict and post-conflict settings must be better understood and addressed in a more equitable manner than is currently the case. The authors of this new study have made a significant contribution to allow us to move forward.

## Abbreviations

CRS: Creditor Reporting System
GFATM: Global Fund to Fight AIDS, Tuberculosis and Malaria
LDC: least-developed country
ODA: official development assistance
RH: reproductive health
UN: United Nations

## References

[1] Inter-Agency Standing Committee (2004). Guidelines for HIV/AIDS in emergency settings.. http://www.unhcr.org/protect/PROTECTION/402c95be4.pdf.

[2] Inter-Agency Standing Committee (2005). Guidelines on gender-based violence interventions in humanitarian settings.. http://www.humanitarianinfo.org/iasc/pageloader.aspx?page=content-subsidi-tf_gender-gbv.

[3] United Nations High Commissioner for RefugeesWorld Health OrganizationUnited Nations Population Fund (1999). Reproductive health in refugee situations: An inter-agency field manual.. http://www.unfpa.org/emergencies/manual/.

[4] McGinn T, Purdin S (2004). Reproductive health and conflict: Looking back and moving ahead.. Disasters.

[5] Patel P, Roberts B, Guy S, Lee-Jones L, Conteh L (2009). Tracking official development assistance for reproductive health in conflict-affected countries.. PLoS Med.

[6] Steering Committee for Humanitarian Response (2004). The Sphere Project: Humanitarian charter and minimum standards in disaster response.. http://www.sphereproject.org/component/option,com_docman/task,cat_view/gid,70/Itemid,203.

[7] Spiegel PB, Bennedsen AR, Claass J, Bruns L, Patterson N (2007). Prevalence of HIV infection in conflict-affected and displaced people in seven sub-Saharan African countries: A systematic review.. Lancet.

[8] Halperin D (2008). Putting a plague in perspective. The New York Times.. http://www.nytimes.com/2008/01/01/opinion/01halperin.html.

[9] Piot P, Kazatchkine M, Dybul M, Lob-Levyt J (2009). AIDS: Lessons learnt and myths dispelled. Lancet..

